# Fatty acid profile in the seeds and seed tissues of *Paeonia* L. species as new oil plant resources

**DOI:** 10.1038/srep26944

**Published:** 2016-05-31

**Authors:** Shuiyan Yu, Shaobo Du, Junhui Yuan, Yonghong Hu

**Affiliations:** 1 Shanghai Key Laboratory of Plant Functional Genomics and Resources, Shanghai Chenshan Plant Science Research Center, Chinese Academy of Sciences, Shanghai Chenshan Botanical Garden, Shanghai 201602, China; 2 East China Normal University, Shanghai 200241, China

## Abstract

Most common plant oils have little α-linolenic acid (C18:3^Δ9,12,15^, ALA) and an unhealthy ω6/ω3 ratio. Here, fatty acids (FAs) in the seeds of 11 species of *Paeonia* L., including 10 tree peony and one herbaceous species, were explored using gas chromatograph–mass spectrometer. Results indicated that all *Paeonia* had a ω6/ω3 ratio less than 1.0, and high amounts of ALA (26.7–50%), oleic acid (C18:1^Δ9^, OA) (20.8–46%) and linoleic acid (C18:2^Δ9,12^, LA) (10–38%). ALA was a dominant component in oils of seven subsection *Vaginatae* species, whereas OA was predominant in two subsection *Delavayanae* species. LA was a subdominant oil component in *P. ostii* and *P. obovata*. Moreover, the FA composition and distribution of embryo (22 FAs), endosperm (14 FAs) and seed coat (6 FAs) in *P. ostii*, *P. rockii* and *P. ludlowii* were first reported. Peony species, particularly *P. decomposita* and *P. rockii*, can be excellent plant resources for edible oil because they provide abundant ALA to balance the ω6/ω3 ratio. The differences in the ALA, LA and OA content proportion also make the peony species a good system for detailed investigation of FA biosynthesis pathway and ALA accumulation.

Tree peony symbolises happiness, wealth and power. It is renowned as Queen Wu Zetian’s favourite during the Tang Dynasty (AD 700), and has been crowned the ‘king of flower’ since that time. Now, tree peony seed oil has attracted considerable attention. The seeds are characterised by abundant unsaturated fatty acid (UFA, >90%) and high proportions of α-linolenic acid (C18:3^Δ9,12,15^, ALA), which accounts for 45%^1^,^2^.

The ω3 and ω6 fatty acids (FAs) are commonly in the form of ALA and linoleic acid (C18:2^Δ9,12^, LA), which are essential FAs for humans and must be obtained in food or dietary supplements^3^. The ω6 and ω3 FAs have reciprocal biological activities, and the ω6/ω3 ratio of consumption is associated with human health^4^. Healthy ratios of ω6 to ω3 FAs should be lower than 5^5^; it has been recommended that human beings evolve on a diet with a ω6/ω3 FA ratio of ∼1^6^. A lack of ω3 FA dietary intake causes a high ratio in our daily diet, such as sunflower (∼670), peanut (∼581.6), corn germ (∼100), olive oil (16.7) and soybean oil (∼3.9)^7^,^8^,^9^, and has been linked to blood lipid, cardiovascular, autoimmune and inflammatory diseases^10^,^11^. Owing to overharvesting and environmental pollution, fish oil can no longer serve as a source of ALA and fish cannot satisfy the vegetarian diet. Currently, plant seed oil, such as perilla (54%), flax (51%), rapeseed (9%), soybean (7%) and walnut (6%)^7^,^12^, has become the major source of ALA. However, as herbaceous plants, perilla and flax promotion is limited because of their low seed production and their competition with food crops^7^. As ALA-enriched resources, tree peony (45%), sacha inchi (50%), sea buckthorn (39%) and cypress (35%) are worthy of further exploration; the tree peony seed oil has a low ω6/ω3 ratio and a potential annual seed production of 57,855 tons^1^,^13^,^14^,^15^. In 2013, tree peony seeds were recommended as a new resource of ALA for seed oil production in China. At the beginning of 2015, the General Office of the State Council of China promulgated the opinions on accelerating the development of the woody oil industry, and tree peony seed oil was clearly proposed. The rediscovery of the value of this oil and the rapid development in recent years have brought unprecedented opportunities and challenges for studies on tree peony.

The genus *Paeonia* is divided into three sections: *Moutan* DC., *Onaepia* Lindl. and *Paeonia*^16^. In China, species in the sections of *Moutan* DC. and *Paeonia* are widely distributed. The former is shrub with a leathery disk that contains paeonol and a small amount of paeoniflorin. The latter is an herbaceous perennial, short and with a fleshy disk, with more paeoniflorin and paeonol-free. According to Hong’s taxonomic^17^, eight wild species, together with two subspecies, constitute section *Moutan* DC., which includes two subsections, namely, *Delavayanae* and *Vaginatae*. Compared with the progress made in *Paeonia* classification, protection, cultivation and origin of cultivated tree peoniy^17^,^18^,^19^,^20^,^21^, our understanding of tree peony oil is limited. To date, the FA compositions in the seeds of 60 tree peony cultivars has been reported, and another study compared the FA compositions in the seed kernel and coat of *P. rockii*^1^,^2^. Our present work aimed to examine the (i) FA composition of various species seeds from the genus *Paeonia* in China, and the (ii) FA distribution in embryo, endosperm and seed coat of *P. ostii*, *P. rockii* and *P. ludlowii*. Such information will be used to evaluate the potential germplasm with amounts of ALA as high-quality edible oil.

## Results

### FA composition in the seeds of various *Paeonia* species

**Figure 1:**
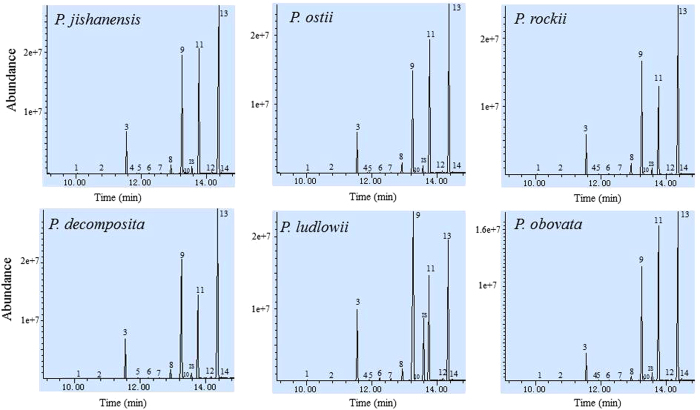
GC–MS of fatty acid methyl esters in the seeds of various *Paeonia* species. 1: C14:0; 2: C15:0; 3: C16:0; 4: C16:1^Δ7^; 5: C16:1^Δ9^; 6: C17:0; 7: C17:1^Δ10^; 8, C18:0; 9: C18:1^Δ9^; 10: C18:1^Δ11^; 11: C18:2^Δ9,12^; 12: C20:0; 13: 18:3^Δ9,12,15^; 14: C20:1^Δ11^; IS: internal standard, nonadecanoic acid.

In the seeds of sample species, 14 FAs were found, with a predominance of ALA (peak 13), LA (peak 11), oleic acid (C18:1^Δ9^, OA, peak 9), stearic acid (C18:0, SA, peak 8) and palmitic acid (C16:0, PA, peak 3) (Fig. 1). Myristic (C14:0, peak 1), pentadecanoic (C15:0, peak 2), cis-7-hexadecenoic (C16:1^Δ7^, peak 4), palmitoleic (C16:1^Δ9^, peak 5), margaric (C17:0, peak 6), heptadecenoic (C17:1^Δ10^, peak 7), vaccenic (C18:1^Δ11^, peak 10), arachidic (C20:0, peak 12) and eicosenoic acids (C20:1^Δ11^, peak 14) were minor FAs. The total FA (TFA) content, which ranged from 109.5 mg g^−1^ to 230.8 mg g^−1^, varied dramatically among the different species (Table 1). The percentage of TFA content was highest in *P. decomposita* (23.1%) and lowest in *P. delavayi* (11%). The saturated FA (SFA) and UFA content among species varied from 10.8 mg g^−1^ to 29.8 mg g^−1^ and from 98.6 mg g^−1^ to 210.8 mg g^−1^ respectively (Table 1). Table 1 also shows that the ratios of ω6/ω3 FA ranged from 0.3 to 0.83 in different species.

The FA compositions of 11 accessions are presented in Table 1. Among the *Paeonia* species, *P. decomposita* ssp. *decomposita* had the highest mean ALA concentration (114.7 mg g^−1^), whereas *P. delavayi* had the lowest (34.71 mg g^−1^). In nine other accessions, *P. decomposita* ssp. *rotundiloba* had the highest ALA concentration (97.54 mg g^−1^), followed by *P. rockii* ssp. *rockii* (90.57–94.47 mg g^−1^) and *P. rockii* ssp. *atava* (87.8 mg g^−1^), *P. jishanensis* (85.51 mg g^−1^), *P. ostii* (83.43 mg g^−1^), cultivars of Japanese tree peony ‘Daojin’ (59.26 mg g^−1^), *P. obovata* (51.99 mg g^−1^) and *P. ludlowii* (40.71 mg g^−1^). *P. ostii* and *P. obovata* had high LA contents at 59.41 and 45.03 mg g^−1^ respectively, whereas *P. delavayi* (11.03 mg g^−1^) had the lowest LA content, followed by *P. ludlowii* (26.93 mg g^−1^). Surprisingly, *P. ludlowii* and *P. delavayi* possessed a higher concentration of OA than ALA, a result that was significantly different from other species. Among used species, *P. ludlowii* (62.58 mg g^−1^) had the highest OA concentration, followed by *P. decomposita* (59.34 mg g^−1^), *P. rockii* (55.7 mg g^−1^) and *P. delavayi* (50.91 mg g^−1^). The lowest OA content was that of *P. obovata* (29.17 mg g^−1^). Furthermore, the minor FA content among tree peony species varied from 2.35 mg g^−1^ to 3.93 mg g^−1^. Unexpectedly, the minor FA content in *P. obovata* was 5.29 mg g^−1^ because its vaccenic acid content was 2.41 mg g^−1^, which was several times more than in other species.

### Variations in the FA percentage content of various *Paeonia* species

**Figure 2:**
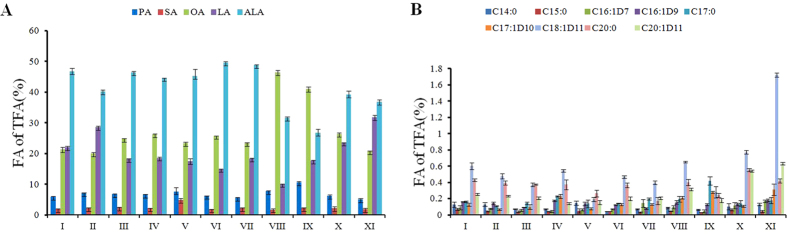
Variations in percentage content of main fatty acids (**A**) and minor fatty acids (**B**) among *Paeonia* species. I: *Paeonia jishanensis*; II: *Paeonia ostii*; III: cultivated *Paeonia rockii* ssp. *rockii*; IV: wild *Paeonia rockii* ssp. *rockii*; V: *Paeonia rockii* ssp. atava; VI: *Paeonia decomposita* ssp. *decomposita*; VII: *Paeonia decomposita* ssp. *rotundiloba*; VIII: *Paeonia delavayi*; IX: *Paeonia ludlowii*; X: Japanese tree peony ‘Daojin’; XI: *Paeonia obovata*.

The results of FA compositions (Fig. 2) revealed significant differences among *Paeonia* species. Five major FAs comprised 97.5–98.7% of TFA content in tree peony species and about 96.2% in *P. obovata* (Fig. 2A). Hence, the minor FAs comprised 3.8% in *P. obovata*, a value higher than that in other tree peony species (2.5–1.3%) (Fig. 2B). On average, as the only major SFAs, the concentrations of PA and SA were basically identical. *P. ludlowii* (SFA, 13.6%; PA, 10.6%), *P. delavayi* (10.3%, 7.8%) and *P. rockii* ssp. *atava* (13.2%, 7.7%) showed much higher percentage contents of SFA and PA than those of other species (Table 1 and Fig. 2A). Particularly for SA, *P. rockii* ssp. *rockii* and *P. rockii* ssp. *atava* showed the highest percentage contents at 2.38% and 4.93% respectively (Fig. 2A). The major UFAs, namely, ALA, OA and LA, varied within 26.7–49.7%, 20.1–46.5% and 10.1–31.9%, respectively (Fig. 2A). Obviously, in most *Paeonia* species, with the exceptions of *P. delavayi* and *P. ludlowii*, ALA was the dominant compound (49.7% in *P. decomposita*), followed by LA and OA. The OA content (46.5%, 41%) was higher than ALA (31.7%, 26.7%) and LA (10.1%, 17.7%) in *P. delavayi* and *P. ludlowii* respectively. Margaric acid (C17:0, MA), which is a rare odd carbon number FA, was also found in all the investigated species. The MA content was higher in *P. ludlowii* (0.42%) than in other species (0.11–0.23%). *P. obovata* had high vaccenic acid (C18:1^Δ11^) content (1.72%), which was much higher than that in other species (0.19–0.78%).

### Wet and dry matter contents of seed tissues

The wet and dry matter contents of 100 seeds and separation tissues (embryo, endosperm and seed coat) of the three species are shown in Table 2. The wet and dry contents of 100 seeds were 26.44 and 22.45 g, 29.57 and 23.58 g and 85.2 and 81.16 g in *P. ostii*, *P. rockii* and *P. ludlowii*, respectively; correspondingly, their seed moisture contents were 15.01%, 20.28% and 4.73%. For the dry matter content, the seeds of *P. ludlowii* exceeded that of *P. ostii* and *P. rockii*, at an average of 3.62- and 3.44-fold respectively. For the dry matter content, the endosperm and seed coat comprised 66.02%, 68.36% and 76.55% and 33.66%, 31.34% and 23.37% of the whole seed weight in *P. ostii*, *P. rockii* and *P. ludlowii*, respectively; correspondingly, the embryo only represented 0.33%, 0.3% and 0.08% of the mature seed dry mass.

### FA composition in the different seed tissues among three species

**Figure 3:**
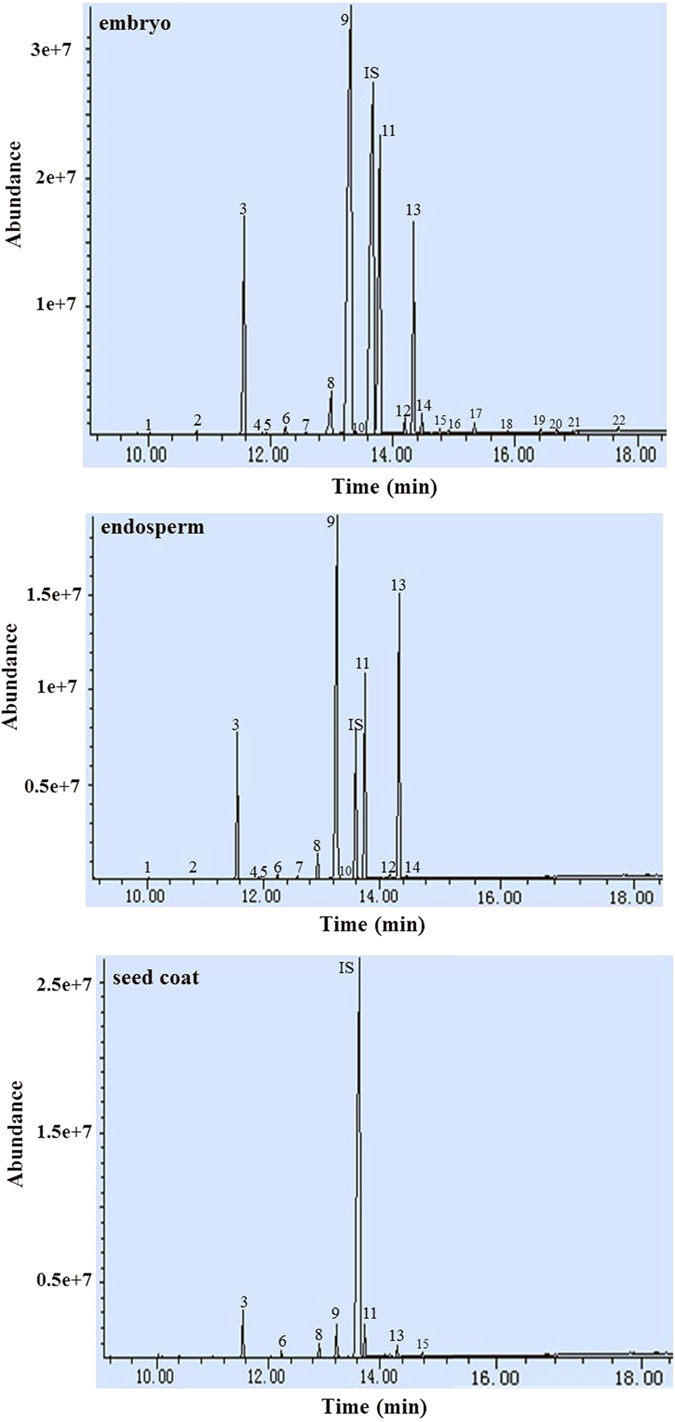
GC–MS of fatty acid methyl esters in embryo, endosperm and seed coat of *Paeonia ludlowii*. 1: C14:0; 2: C15:0; 3: C16:0; 4: C16:1^Δ7^; 5: C16:1^Δ9^; 6: C17:0; 7: C17:1^Δ10^; 8, C18:0; 9: C18:1^Δ9^; 10: C18:1^Δ11^; 11: C18:2^Δ9,12^; 12: C20:0; 13: 18:3^Δ9,12,15^; 14: C20:1^Δ11^; 15: C21:0; 16: C20:2^Δ11,14^; 17: C22:0; 18: C22:1^Δ13^; 19: C23:0; 20: C24:0; 21: C24:1^Δ15^; 22: C25:0; IS: internal standard, nonadecanoic acid.

Figure 3 and Table 3 show that the tissues displayed high variation for FA composition in tree peony seeds. For *P. ludlowii*, a total of 22, 14 and 7 FAs were found respectively in embryo, endosperm and seed coat. Identical results were obtained for *P. ostii* and *P. rockii*, except that only 6 FAs were observed in their seed coat and 19 FAs were found in *P. rockii* embryo. As the minor FAs, heneicosanoic (C21:0, peak 15), eicosadienoic (C20:2^Δ11,14^, peak 16), behenic (C22:0, peak 17), erucic (C22:1^Δ13^, peak 18), tricosanoic (C23:0, peak 19), tetracosanoic (C24:0, peak 20), tetracosaenoic (C24:1^Δ15^, peak 21) and pentacosanoic acids (C25:0, peak 22) were only detected in embryo, but not in endosperm, and only one (C21:0) in the *P. ludlowii* seed coat. The TFA content varied dramatically among different tissues of *P. ostii*, *P. rockii* and *P. ludlowii* at 264.3, 199.5 and 238.4 mg g^−1^ in embryo, 342.1, 271.1 and 174.1 mg g^−1^ in endosperm and 1.61, 3.46 and 4.13 mg g^−1^ in seed coat, respectively (Table 3).

In *P. ostii*, LA (137.7 mg g^−1^) predominated in embryo oil, and OA (51.67 mg g^−1^) and ALA (50.98 mg g^−1^) were subdominant. The major FAs of endosperm oil were ALA (144.1 mg g^−1^), LA (107.7 mg g^−1^) and OA (66.21 mg g^−1^). However, the oil and FA in seed coat were present in small quantities, and PA predominated FA at 0.57 mg g^−1^. In *P. rockii*, LA (63.2 mg g^−1^) still predominated in embryo oil, followed by ALA (58.19 mg g^−1^) and OA (56.72 mg g^−1^). ALA was present at the largest level in endosperm (148.2 mg g^−1^), followed by OA (59.61 mg g^−1^) and LA (45.73 mg g^−1^). For seed coat, FA had a trace amount, and PA predominated at 1.25 mg g^−1^. However, in *P. ludlowii*, OA was predominant not only in embryo (122.1 mg g^−1^) but also in endosperm (70.64 mg g^−1^). PA (2.06 mg g^−1^) was predominant in seed coat. The ω6/ω3 ratios also varied in different tissues, ranging within 1.1–2.7 in embryo, 0.3–0.7 in endosperm and 2–2.3 in seed coat (Table 3).

### Characteristics of major FA content in embryo, endosperm and seed coat

**Figure 4:**
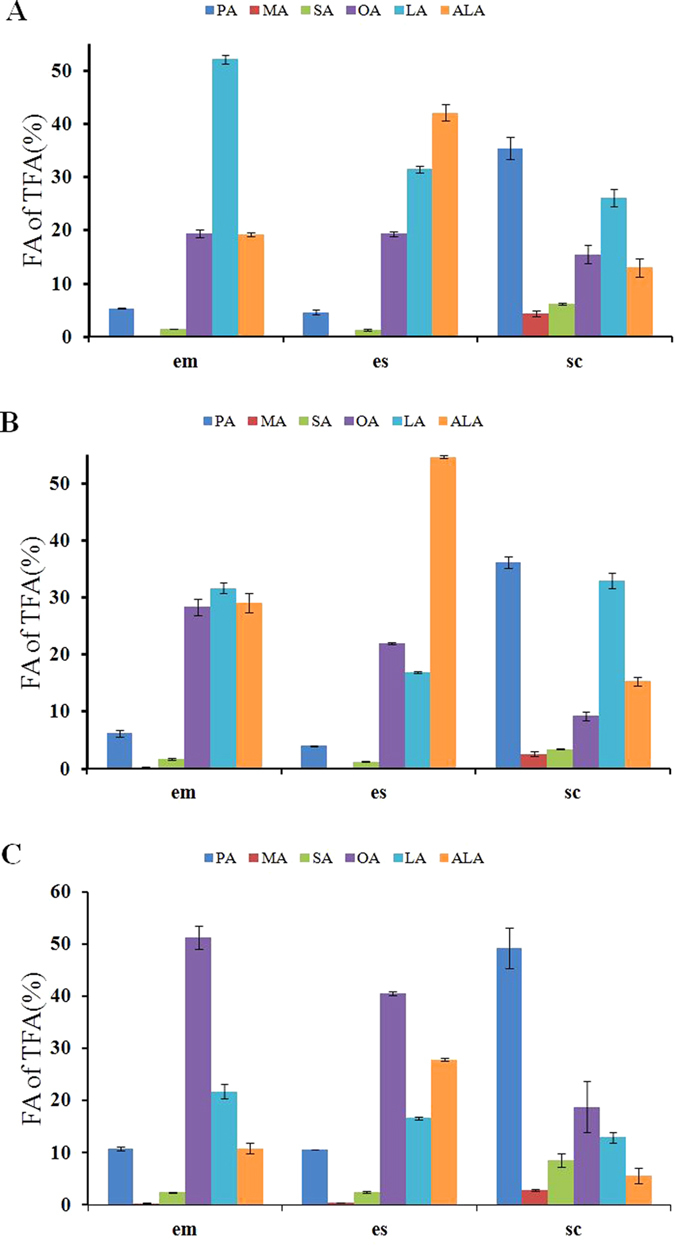
Variations in percentage content of main fatty acids in embryo (em), endosperm (es) and seed coat (sc) of different species. (**A**) *Paeonia ostii*; (**B**) *Paeonia rockii*; (**C**) *Paeonia ludlowii*. PA: palmitic acid, C16:0; MA: margaric acid, C17:0; SA: stearic acid, C18:0; OA: oleic acid, C18:1^Δ9^; LA: linoleic acid, C18:2^Δ9,12^; ALA: α-linolenic acid, C18:3^Δ9,12,15^.

Figure 4 presents the range and distribution of PA, MA, SA, OA, LA and ALA percentage contents in embryo, endosperm and seed coat of the three species. The content of six dominant FAs varied significantly in different tissues. The major SFAs, namely, PA, MA and SA, comprised 60.5% of the TFA content of seed coat in *P. ludlowii*, 46% in *P. ostii* and 42.2% in *P. rockii*. However, the SFA content of embryo and endosperm was lower than that of seed coat. For *P. ludlowii*, *P. ostii* and *P. rockii*, the SFA content was only 13.4%, 7.0% and 8.2% of embryo, and 13.4%, 6.2% and 5.5% of endosperm, respectively. Correspondingly, the contents of major UFAs, namely, OA, LA and ALA, were higher in embryo and endosperm than that in the seed coat of the three species. ALA was the dominant compound in *P. ostii* and *P. rockii* endosperm at 42.1% and 54.7% respectively, but *P. ludlowii* was a distinct species with higher OA (40.6%) content than ALA (27.8%) in endosperm. Furthermore, the OA (51.3%) was still a dominant compound in *P. ludlowii* embryo, and LA was subdominant. In *P. ostii* and *P. rockii* embryos, LA predominated at 52.2% and 31.7, and OA and ALA were the subdominant compounds at 19.5% and 19.3%, and 28.4% and 29.1% respectively. Obviously, PA was an identical dominant compound in the seed coat of *P. ostii*, *P. rockii* and *P. ludlowii* at 35.4%, 36.1% and 49.2%, respectively.

## Discussion

In the present study, 14 FAs were found in the seeds of all examined species. A total of 22 kinds of FA were also detected in the embryos of *P. ludlowii*, *P. ostii* and *P. rockii*. However, Li *et al.*^1^ reported only nine kinds of FA in the seeds of 60 tree peony cultivars. In their study, the five dominant FAs were identical to our present research, but high variability was observed in the quantitative results. In addition, there are many significant minor FAs detected in our study. MA is one of the most important FAs found in all detected *Paeonia* species, specifically in the seed coat of three species (Table 3 and Fig. 4). In the previous study, MA was not detected maybe because methyl heptadecanoate was employed as the internal standard^1^,^22^. However, Li *et al.*^2^ reported the presence of MA in the seed kernel and coat of *P. rockii*. Some differences were also observed in the FA content in various studies. The differences could be attributed to the quantitative analysis method, different cultivars, growing conditions, harvest time and storage method. For the measurement of FA content, FA (mg)/seed dry weight (DW, g) is a typical unit of measure, which was the first option used in our study and in other research^1^,^22^. Even in the same research group, the FA compositions and contents achieved in the two studies were not completely identical^1^,^22^. Trace amounts of minor FA were nearly equal in all examined species, and only vaccenic acid (C18:1^Δ11^) was detected at a high level in herbaceous *P*. *obovata*. The seed of *P*. *obovata* had more than 8.6-, 6.1- and 3.6-fold the amount of vaccenic acid than in the seed of *P. rockii* ssp. atava, *P. ludlowii* and *P. ostii*, respectively (Fig. 2B). This result was a significant characteristic between herbaceous and tree peonies.

*P. ludlowii* and *P*. *delavayi* belong to subsection *Delavayanae*, whereas *P*. *jishanensis*, *P*. *ostii*, *P*. *rockii* and *P*. *decomposita* belong to subsection *Vaginatae*. Evidently, the FA composition and content of the two subsections were significantly different, particularly for major UFA. The seed oils of all examined species from subsection *Vaginatae* were richer in ALA than in LA and OA. Surprisingly, the subsection *Delavayanae* species possessed a higher OA concentration than ALA and LA. Thus, *Delavayanae* members could be a *Vaginatae* control study system for the FA desaturase (FAD), specifically for Δ15-desaturase. In subsection *Vaginatae*, the species also exhibited different FA composition characteristics. LA was present as a subdominant compound in *P. ostii* and *P. jishanensis*, and OA was subdominant in *P*. *rockii* and *P*. *decomposita*. Therefore, the two major UFA proportions in the seed oil of subsection *Vaginatae* species were not identical. On the contrary, for the seed oil of subsection *Delavayanae* species, OA was dominant, ALA was subdominant and LA was a third compound, findings that were consistent within *P*. *ludlowii* and *P*. *delavayi*. ALA was also the dominant compound, and LA was subdominant in *P*. *obovata* seed oil, which belonged to section *Paeonia*. This major FA composition proportion in *P*. *obovata* was identical to that in *P. ostii* and *P. jishanensis*.

This research firstly reported the FA composition and content of seed in *P*. *decomposita*. The Danba population of *P. decomposita* ssp. *decomposita*, which is located within the altitude of 2050–3000 m, and Lixian population of *P. decomposita* ssp. *rotundiloba*, which is located within the altitude of 1700–2700 m, were sampled in this study. Obviously, the ALA and TFA contents in *P*. *decomposita* ssp. *decomposita* were significantly the highest, followed by those of *P*. *decomposita* ssp. *rotundiloba* for ALA concentration. Except for *P. decomposita*, *P. rockii* showed the second higher levels of TFA and ALA contents among investigated species. Wild-type *P. rockii* ssp. *rockii* from Longguliang population, cultivated *P. rockii* from Yuzhong population and *P. rockii* ssp. *atava* from Taibai population were sampled to evaluate the seed oil quality. Wild species had higher TFA and ALA contents than cultivated species. By contrast, the wild-type ssp. *atava* showed lower levels of TFA and ALA concentrations than the wild-type ssp. *rockii* and cultivated *P. rockii*. Among investigated species, *P. jishanensis* exhibited the third higher levels of TFA and ALA contents. The wild populations of *P. decomposita*, *P. rockii* and *P. jishanensis* were all grown within high altitude areas of 2050–3000, 1100–2800 and 900–700 m, respectively^17^,^18^. Therefore, the large amount of ALA accumulation was closely related to the ecological environment of the plant, particularly the high altitude. Recently, Connor *et al.*^23^ reported that FA composition may be too sensitive to environmental conditions to be a reliable signal of physiological maturity. Rapeseed oil content is a typical quantitative trait that can be easily influenced by environmental factors^24^. In a soybean oil study, the ALA concentration of wild soybean accessions was correlated with the growing environment^25^.

The FA compositions and distributions of embryo, endosperm and seed coat in *P. ostii*, *P. rockii* and *P. ludlowii* were reported in the present study for the first time. The FA composition and content in seed tissues were dramatically different. Because the low oil content of seed coat and the low dry weight of embryo, the endosperm had the highest oil yield. PA was the dominating compound in the seed coat, which was identical in the three species. For *P*. *ostii* and *P. rockii*, ALA was the dominant compound in endosperm, and LA was predominant in embryo. But for the embryo and endosperm in *P*. *ludlowii*, the FA profile was dominated by OA. Different seeds tissues also display high variation for FA composition, e.g. coffee seed, argan seed, and oil palm^26^,^27^,^28^. The tissue-specific gene expression or correlated transcription factor results in various FA distributions^28^. The sugar content in seed coat is correlated significantly and positively with rapeseed oil content, and seed coat may affect oil synthesis in embryo by regulating sucrose transport^29^. Thus, the difference in the FA composition of different tissues has many possible causes and is regulated by biochemical factor and genetic manipulation.

ALA is the shorter-chain ω3 FA from plant oil, and its deficiency could lead to abnormal function^30^,^31^. A high ω6/ω3 ratio in the diet can accentuate ω3 FA deficiency, e.g. edible oil rich in corn germ (∼100), peanut (∼581.6) and sunflower (∼670)^7^. The optimal ratio may vary with the disease under consideration: 5:1 ratio can suppress asthma symptoms, 4:1 is good for the prevention of cardiovascular disease and 2.5:1 suppresses colon cancer cell proliferation^6^. Another report suggested that the ideal ratio of ω6 to ω3 FA is 1:1–2:1^32^. Surprisingly, the ω6/ω3 ratio in peony seed oil is less than 1.0 in all analysed species. The lowest ratio was nearly 0.3 in the seed oil of *P. decomposita* ssp. *decomposita* and *P. delavayi*, followed by 0.4 of *P. decomposita* ssp. *rotundiloba*, *P. rockii* ssp. *rockii* and *P. rockii* ssp. *atava*. The highest ratio was only 0.8 in the seed oil of *P. obovata*, followed by 0.7 of *P. ostii* and *P. ludlowii*. Interestingly, the ω6/ω3 ratio of endosperm (0.3, 0.6) was lower than that of whole seed (0.4, 0.7) in *P. rockii* and *P. ludlowii*, but the ratio was nearly 0.8 of endosperm in *P. ostii*, which was higher than that of whole seed (0.7). The endosperm is the main storage of tree peony seed oil, and thus had the highest ALA and LA content proportions.

In summary, this study evaluated the seed oil quality of 11 species of *Paeonia* L. All *Paeonia* species had less than 1.0 ω6/ω3 ratio. The species also had dramatically high ALA content, ranging from 26.7% (*P. ludlowii*) to 50% (*P. decomposita* ssp. *decomposita*); relatively high content of OA ranging from 20.8% (*P. ostii*) to 46% (*P. ludlowii*); and high content of LA ranging from 10% (*P. delavayi*) to 38% (*P. obovate*). The FA composition in the seed oil of *P. decomposita*, which had the highest ALA content, was firstly reported. Interestingly, a lower ALA was generally observed in the two subsection *Delavayanae* species of *P. ludlowii* and *P. delavayi* than in other subsection *Vaginatae* species. On the contrary, a higher OA was often found in *Delavayanae* than in *Vaginatae*. OA was a dominant compound in *Delavayanae*, whereas ALA was predominant in *Vaginatae* species. LA was a subdominant compound in the seed oil of *P. ostii* and *P. obovata*, which was different from OA being a subdominant compound in *P. rockii* and *P. decomposita*. Moreover, the FA composition and distribution of embryo, endosperm and seed coat in *P. ostii*, *P. rockii* and *P. ludlowii* were also reported for the first time. Embryo had 22 FAs, endosperm had 14 FAs and only 6 FAs were found in seed coat. Taken together, peony species, particularly *P. decomposita* and *P. rockii*, had high seed production, high ALA content and low ω6/ω3 ratio, and would thus be an excellent resource in providing abundant ALA edible oil. Furthermore, *P. decomposita*, *P. ostii*, *P. obovata*, *P. delavayi* and *P. ludlowii* differed in ALA, LA and OA content proportions. Hence, the peony species will be a good study system for the mechanism of FA metabolism and ALA accumulation. However, as a good source of ALA and other PUFAs, peony oil could be more hypersensitive to lipid peroxidation processes, especially in the industrial production. Lipid peroxidation process might be a more complicated problem, and this effect will be investigated in the future work step by step.

## Methods

### Seed materials

The *Paeonia* species used in this study were *P. jishanensis*, *P. ostii*, *P. rockii* ssp. *rockii* and ssp. atava, *P. decomposita* ssp. *decomposita* and ssp. *rotundiloba*, *P. delavayi*, *P. ludlowii*, *P. obovata* and one cultivar of Japanese tree peony ‘Daojin’. The plants were introduced into the garden and cultivated for three years at Shanghai Chenshan Botanical Garden (31°4′52″N, 121°10′14″E). In August 2015, the seeds were harvested when the plants had attained complete physiological maturity, and each seed sample was obtained from three to four plants of a given species. The mature seeds were used for FA analysis and stored in brown headspace vials filled with nitrogen. Independent samples were analysed in triplicate.

### Separation of seed coat, endosperm and embryo

A total of 373, 200 and 210 matured seeds were directly hand-picked from *P. ostii*, *P. rockii* and *P. ludlowii*, respectively. Subsequently, the seed coat, endosperm and embryo of each seed were separated, weighed and flash-frozen in liquid nitrogen. All the separated embryos were used for the FA analysis. Seed coat and endosperm were randomly sampled from three to five seeds.

### Extracting lipids

Stored seeds were dried to a constant weight at 60 °C. Dried seeds comprising three replicate samples were pulverised in liquid nitrogen using a mortar and pestle. The total lipid content was extracted from the biomass according to Folch *et al.*^33^ with slight modification. Firstly, approximately 0.2 g of dried powder was weighed and placed in 20 mL screw-cap glass tubes, in which 3 mL of 1:2 chloroform:methanol (v/v) mixture was added. The extraction was performed in a water bath at 35 °C for 1 h at a rotation speed of 120 rpm. After full extraction, 1.0 mL of supplementary chloroform was added to the solution, which was then re-vortexed. Next, 1.8 mL ddH_2_O was added so that the final volume ratio of chloroform:methanol:ddH_2_O was 1:1:0.9. This process was followed by centrifugation at 4000 g for 15 min. The chloroform layer was collected and transferred to another 20 mL screw-cap glass tube. The entire extraction process was repeated twice. After phase separation, the chloroform layer was withdrawn, dried with sample concentrators under a nitrogen evaporator and stored at −20 °C for further use.

### FA methylation

FA methylation was prepared according to the procedures described by Bligh and Dyer^34^. The concentrated seed lipids prepared above were re-dissolved in 2 mL H_2_SO_4_ methanol solution (4% H_2_SO_4_). After charging with nitrogen gas, the bottle was vortexed for 1 min and placed in 90 °C water bath for 1 h. The bottle was mixed by vortexing after the addition of 1 mL ddH_2_O and 1 mL hexane, followed by centrifugation at 4000 g for 15 min. The supernatant was transferred to a new tube, concentrated by bubbling nitrogen and stored in 4 °C for GC-MS analysis. Additionally, 20 μL nonadecanoic acid (50 mg/mL in hexane) was used as the internal standard for each sample.

### FA analysis

The FA methyl esters were subjected to GC–MS (GC7890/MS5975, Agilent) on a HP-88 capillary column (60 m × 0.25 mm, 0.2 μm, Agilent). The column temperature was held at 70 °C for 1 min, increased to 210 °C at 10 °C/min for 0 min and then to 220 °C at 10 °C/min for 0 min, and subsequently to 235 °C at 10 °C/min for 8 min. The injector temperature was set at 250 °C for split injection at a split ratio of 5:1. The injection volume was 1 μL. Helium was used as the carrier gas at a flow rate of 1 mL/min, and the ionisation potential of the mass-selective detector was 70 eV.

FA identification was achieved through a mass spectrum database search (NIST MS Search 2.0) and co-eluted with the 37-component FAME Mix (Sigma, USA). A standard curve method with an internal standard was used as the quantitative approach to construct three calibration plots of internal standard peak–area ratio versus standard concentration, as determined by the least squares method. The five major FAs in each sample were quantified in absolute terms using the linear regression of their corresponding standard, while the minor FAs were measured using methyl nonadecanoic acid as the internal standard. FAMEs were expressed as milligrams per gram DW of sample. All samples were analysed in triplicate.

### Statistical analysis

The statistical analysis method was used for data processing according to the procedures described by Dunn and Clark^35^. All experiments were performed with three replicates. The statistical analysis of the FA contents and percentages were tested by One-way ANOVA analysis (p < 0.05) and comparisons between means were performed with Tukey′s test.

## Additional Information

**How to cite this article**: Yu, S. *et al.* Fatty acid profile in the seeds and seed tissues of *Paeonia* L. species as new oil plant resources. *Sci. Rep.* **6**, 26944; doi: 10.1038/srep26944 (2016).

## Figures and Tables

**Table 1:** Fatty acid composition in the seeds of various *Paeonia* species (mg/g dry weight of seed).

FAs	I	II	III	IV	V	VI	VII	VIII	IX	X	XI
C16:0	10.92 ± 0.43	14.55 ± 0.88	13.30 ± 1.1	13.86 ± 0.38	14.88 ± 1.18	14.24 ± 1.35	11.23 ± 0.71	8.53 ± 0.92	16.15 ± 1.79	9.36 ± 0.79	7.02 ± 0.39
C18:0	3.53 ± 0.07	4.67 ± 0.39	4.65 ± 0.36	4.54 ± 0.28	9.56 ± 1.51	4.40 ± 0.48	4.55 ± 0.23	1.96 ± 0.10	3.47 ± 0.52	3.39 ± 0.10	2.67 ± 0.08
C18:1^Δ9^	39.01 ± 3.22	41.59 ± 2.49	48.35 ± 4.45	55.70 ± 2.87	45.23 ± 2.88	59.34 ± 1.87	46.90 ± 4.32	50.91 ± 1.26	62.58 ± 1.94	39.92 ± 0.78	29.17 ± 3.56
C18:2^Δ9,12^	40.30 ± 1.26	59.41 ± 1.51	35.61 ± 1.35	39.42 ± 2.29	34.01 ± 1.76	34.49 ± 1.39	36.81 ± 1.57	11.03 ± 1.54	26.93 ± 2.84	35.55 ± 1.06	45.03 ± 1.07
C18:3^Δ9,12,15^	85.51 ± 1.53	83.43 ± 2.27	90.57 ± 2.47	94.47 ± 4.76	87.80 ± 2.04	114.70 ± 1.18	97.54 ± 3.81	34.71 ± 4.45	40.71 ± 4.14	59.26 ± 0.54	51.99 ± 3.05
C14:0	0.23 ± 0.04	0.27 ± 0.03	0.14 ± 0.01	0.14 ± 0.007	0.29 ± 0.04	0.10 ± 0.005	0.14 ± 0.01	0.09 ± 0.004	1.00 ± 0.007	0.15 ± 0.03	0.18 ± 0.01
C15:0	0.13 ± 0.02	0.09 ± 0.01	0.08 ± 0.01	0.09 ± 0.01	0.10 ± 0.01	0.11 ± 0.01	0.06 ± 0.006	0.05 ± 0.004	0.05 ± 0.008	0.10 ± 0.02	0.06 ± 0.01
C16:1^Δ7^	0.16 ± 0.03	0.16 ± 0.01	0.12 ± 0.03	0.09 ± 0.004	0.13 ± 0.02	0.16 ± 0.01	0.28 ± 0.01	0.10 ± 0.009	0.07 ± 0.01	0.17 ± 0.01	0.23 ± 0.01
C16:1^Δ9^	0.29 ± 0.01	0.30 ± 0.01	0.18 ± 0.02	0.37 ± 0.003	0.28 ± 0.01	0.28 ± 0.02	0.16 ± 0.01	0.17 ± 0.01	0.20 ± 0.02	0.20 ± 0.02	0.26 ± 0.02
C17:0	0.30 ± 0.02	0.22 ± 0.00	0.28 ± 0.01	0.49 ± 0.02	0.27 ± 0.01	0.33 ± 0.009	0.39 ± 0.01	0.21 ± 0.002	0.64 ± 0.01	0.22 ± 0.01	0.25 ± 0.007
C17:1^Δ10^	0.23 ± 0.01	0.14 ± 0.003	0.19 ± 0.006	0.49 ± 0.01	0.16 ± 0.01	0.29 ± 0.005	0.26 ± 0.01	0.23 ± 0.008	0.43 ± 0.01	0.17 ± 0.02	0.43 ± 0.004
C18:1^Δ11^	1.10 ± 0.20	0.99 ± 0.01	0.73 ± 0.01	1.16 ± 0.02	0.39 ± 0.01	1.08 ± 0.03	0.79 ± 0.02	0.71 ± 0.004	0.42 ± 0.01	1.17 ± 0.02	2.41 ± 0.01
C20:0	0.79 ± 0.01	0.82 ± 0.003	0.73 ± 0.01	0.79 ± 0.02	0.54 ± 0.01	0.85 ± 0.01	0.34 ± 0.006	0.44 ± 0.002	0.37 ± 0.006	0.84 ± 0.01	0.59 ± 0.01
C20:1^Δ11^	0.47 ± 0.004	0.48 ± 0.01	0.41 ± 0.01	0.30 ± 0.004	0.31 ± 0.006	0.46 ± 0.01	0.41 ± 0.02	0.34 ± 0.002	0.28 ± 0.006	0.82 ± 0.02	0.88 ± 0.01
TFA amounts	183	207.1	195.3	212	194	230.8	200.2	109.5	152.6	151.3	141.2
% of TFA	18.3	20.7	19.5	21.2	19.4	23.1	20.0	11.0	15.3	15.1	14.1
SFA amounts	15.9	20.5	19.1	20	25.7	20	16.7	10.9	20.8	14	10.8
%SFA of TFA	8.7	10	9.8	9.4	13.2	8.7	8.4	10.3	13.6	9.3	7.7
UFA amounts	167.1	186.6	176.2	192	168.3	210.8	183.5	98.6	131.8	137.3	130.4
%UFA of TFA	91.3	90	90.2	90.6	86.8	91.3	91.6	89.7	86.4	90.7	92.3
ω6/ω3	0.5	0.7	0.4	0.4	0.4	0.3	0.4	0.3	0.7	0.6	0.8

FAs: fatty acids; UFA: unsaturated fatty acid; I: *Paeonia jishanensis*; II: *Paeonia ostii*; III: cultivated *Paeonia rockii* ssp. *rockii*; IV: wild *Paeonia rockii* ssp. *rockii*; V: *Paeonia rockii* ssp. *atava*; VI: *Paeonia decomposita* ssp. *decomposita*; VII: *Paeonia decomposita* ssp. *rotundiloba*; VIII: *Paeonia delavayi*; IX: *Paeonia ludlowii*; X: Japanese tree peony ‘Daojin’; XI: *Paeonia obovata*.

**Table 2:** Wet and dry weights of 100 seeds, embryo, endosperm and seed coat.

			*Paeonia ostii*	*Paeonia rockii*	*Paeonia ludlowii*
Wet Weight	Seed Weight (g)		26.44 ± 1.50	29.57 ± 2.27	85.20 ± 0.98
	Embryo	weight (g)	0.0826 ± 0.005	0.0819 ± 0.003	0.0742 ± 0.003
		of seed (%)	0.31	0.28	0.08
	Endosperm	weight (g)	17.29 ± 1.11	20.54 ± 1.75	64.78 ± 0.84
		of seed (%)	65.41	69.46	76.09
	Seed Coat	weight (g)	9.06 ± 0.39	8.95 ± 0.53	20.35 ± 0.26
		of seed (%)	34.28	30.26	23.83
Dry Weight	Seed Weight (g)		22.45 ± 0.77	23.58 ± 1.93	81.16 ± 0.83
	Embryo	weight (g)	0.0739 ± 0.0001	0.0699 ± 0.0027	0.0657 ± 0.001
		of seed (%)	0.33	0.30	0.08
	Endosperm	weight (g)	14.82 ± 0.64	16.12 ± 1.43	62.07 ± 0.70
		of seed (%)	66.02	68.36	76.55
	Seed Coat	weight (g)	7.56 ± 0.13	7.39 ± 0.50	19.02 ± 0.25
		of seed (%)	33.66	31.34	23.37
Seed Moisture Content (%)			15.01 ± 1.86	20.28 ± 1.04	4.73 ± 0.85

**Table 3:** Fatty acid contents in the embryo (em), endosperm (es) and seed coat (sc) (mg/g tissue dry weight).

	*Paeonia ostii*			*Paeonia rockii*			*Paeonia ludlowii*		
	em	es	sc	em	es	sc	em	es	sc
C16:0	14.09 ± 1.47	15.87 ± 0.65	0.57 ± 0.1	12.25 ± 1.92	10.88 ± 1.49	1.25 ± 0.08	25.61 ± 1.83	18.41 ± 1.49	2.06 ± 0.54
C18:0	4.09 ± 0.45	4.72 ± 0.35	0.10 ± 0.04	3.36 ± 0.44	3.43 ± 0.46	0.12 ± 0.01	5.67 ± 0.46	4.25 ± 0.48	0.34 ± 0.02
C18:1^Δ9^	51.67 ± 6.62	66.21 ± 2.10	0.25 ± 0.07	56.72 ± 14.21	59.61 ± 9.51	0.32 ± 0.16	122.1 ± 9.4	70.64 ± 3.45	0.75 ± 0.02
C18:2^Δ9,12^	137.7 ± 11.14	107.7 ± 4.75	0.42 ± 0.08	63.20 ± 14.86	45.73 ± 6.9	1.14 ± 0.35	51.83 ± 4.44	28.91 ± 5.13	0.54 ± 0.14
C18:3^Δ9,12,15^	50.98 ± 5.54	144.1 ± 13.0	0.21 ± 0.01	58.19 ± 15.43	148.2 ± 23.97	0.53 ± 0.11	25.69 ± 3.03	48.54 ± 3.61	0.24 ± 0.09
C14:0	0.12 ± 0.007	0.13 ± 0.01	NA	0.13 ± 0.04	0.14 ± 0.01	NA	0.16 ± 0.02	0.11 ± 0.02	NA
C15:0	0.20 ± 0.01	0.11 ± 0.01	NA	0.20 ± 0.05	0.11 ± 0.01	NA	0.29 ± 0.03	0.06 ± 0	NA
C16:1^Δ7^	0.07 ± 0.001	0.08 ± 0.001	NA	0.11 ± 0.001	0.14 ± 0.03	NA	0.06 ± 0.009	0.07 ± 0.008	NA
C16:1^Δ9^	0.24 ± 0.06	0.34 ± 0.03	NA	0.28 ± 0.12	0.31 ± 0.03	NA	0.12 ± 0.01	0.24 ± 0.04	NA
C17:0	0.39 ± 0.00	0.39 ± 0.09	0.07 ± 0.001	0.44 ± 0	0.52 ± 0.14	0.09 ± 0.001	0.73 ± 0.06	0.70 ± 0.03	0.11 ± 0.002
C17:1^Δ10^	0.22 ± 0.06	0.36 ± 0.05	NA	0.17 ± 0.08	0.36 ± 0.06	NA	0.22 ± 0.02	0.50 ± 0.07	NA
C18:1^Δ11^	0.46 ± 0.02	0.67 ± 0.03	NA	0.93 ± 0.26	0.42 ± 0.16	NA	0.46 ± 0.16	0.58 ± 0.14	NA
C20:0	0.59 ± 0.03	0.49 ± 0.02	NA	0.67 ± 0.18	0.46 ± 0.09	NA	1.22 ± 0.2	0.56 ± 0.06	NA
C20:1^Δ11^	0.66 ± 0.03	0.41 ± 0.02	NA	0.77 ± 0.21	0.30 ± 0.11	NA	1.56 ± 0.3	0.34 ± 0.04	NA
C21:0	0.06 ± 0.002	NA	NA	0.09 ± 0.01	NA	NA	0.32 ± 0.07	NA	0.09 ± 0.007
C20:2^Δ11,14^	0.05 ± 0.03	NA	NA	0.08 ± 0.09	NA	NA	0.27 ± 0.04	NA	NA
C22:0	1.67 ± 0.14	NA	NA	1.02 ± 0.04	NA	NA	0.75 ± 0.07	NA	NA
C22:1^Δ13^	0.07 ± 0.005	NA	NA	NA	NA	NA	0.11 ± 0.02	NA	NA
C23:0	0.15 ± 0.004	NA	NA	0.15 ± 0.03	NA	NA	0.14 ± 0.04	NA	NA
C24:0	0.31 ± 0.03	NA	NA	0.21 ± 0.06	NA	NA	0.34 ± 0.05	NA	NA
C24:1^Δ15^	0.08 ± 0	NA	NA	NA	NA	NA	0.27 ± 0.03	NA	NA
C25:0	0.06 ± 0.006	NA	NA	NA	NA	NA	0.31 ± 0.07	NA	NA
TFA	264.3	342.1	1.61	199.5	271.1	3.46	238.4	174.1	4.13
% of TFA	26.4	34.2	0.18	20.0	27.1	0.37	23.8	17.4	0.41
SFA amounts	21.73	21.62	0.74	18.52	15.54	1.46	35.54	24.09	2.6
%SFA of TFA	8.2	6.3	46	9.3	5.7	42.2	14.9	13.8	63
UFA amounts	242.57	320.48	0.87	180.98	255.56	2	202.86	150.1	1.53
%UFA of TFA	91.8	93.7	54	90.8	94.3	57.8	85.1	86.2	37.1
ω6/ω3	2.7	0.8	2	1.1	0.3	2.2	2	0.6	2.3
